# Valorization of Hemp, Shrimp and Blue Crab Co-Products as Novel Culture Media Ingredients to Improve Protein Quality and Antioxidant Capacity of Cultured Meat in Cell-Based Food Applications

**DOI:** 10.3390/foods15020352

**Published:** 2026-01-18

**Authors:** Davide Lanzoni, Simona Manuguerra, Rosaria Arena, Andrea Santulli, Luca Marchetti, Concetta Maria Messina, Carlotta Giromini

**Affiliations:** 1 Department of Veterinary Medicine and Animal Science, University of Milan, Via dell’Università 6, 26900 Lodi, Italy; davide.lanzoni@unimi.it (D.L.); luca.marchetti1@unimi.it (L.M.); 2 Marine Biochemistry and Ecotoxicology Laboratory, Department of Earth and Marine Science DiSTeM, University of Palermo, Via G. Barlotta 4, 91100 Trapani, Italy; simona.manuguerra@unipa.it (S.M.); rosaria.arena@unipa.it (R.A.); andrea.santulli@unipa.it (A.S.); 3 Institute for Food, Nutrition and Health, University of Reading, Reading RG6 5EU, UK

**Keywords:** cell culture media, cultured meat, proliferation, serum free media, sustainability

## Abstract

Cultured meat (CM) is a promising alternative to conventional livestock production. However, its scalability is limited by the reliance on fetal bovine serum (FBS) in cell culture media (CCM). This study investigated protein hydrolysates derived from hemp flowers (HFs), hempseeds (HSs), hempseed protein (HP), shrimp (SH), and blue crab (BC) co-products as sustainable CCM supplements. Hydrolysates were produced by Alcalase^®^ enzymatic hydrolysis and tested on C2C12 murine myoblasts proliferation and viability. At the concentration of 11.7 mg/mL, no significant differences in cell viability were observed between hydrolysates and 10% FBS at 24 and 48 h. At 72 h post-treatment, 10% FBS resulted in the greatest increase in cell proliferation, whereas SH and BC treatments preserved a more physiological myoblastic morphology. Intracellular protein accumulation at 72 h in 10% FBS- and SH-treated cells was equal to 24.66 ± 1.37 and 18.79 ± 1.99 µg/mg, respectively, and 5.75 ± 2.32 µg/mg in BC while hemp-derived hydrolysates exhibited limited intracellular protein utilization. All hydrolysates significantly enhanced intracellular antioxidant activity compared with FBS (5.83 ± 1.12 µmol FeSO_4_/mg). Although further studies are required to assess long-term performance and large-scale applicability, these findings demonstrate the short-term potential of plant- and marine-derived co-products as sustainable CCM supplements, particularly for enhancing the antioxidant profile of cell biomass.

## 1. Introduction

The rapid evolution of cellular agriculture is reshaping the landscape of sustainable food system, offering innovative strategies to complement traditional livestock production with alternative cell-based systems [1]. Among these, cultured meat (CM) represents an innovative food technology with the potential to reduce environmental impact [2], minimize ethical issues related to animal welfare [3], and improve food security [4]. Beyond these advantages, a general and still unmet goal of CM is not only to provide an alternative protein source, but also to produce meat with an enhanced nutritional profile, in particular in terms of protein content, and added functional properties that could benefit consumer health.

A major bottleneck in CM production is the cell culture medium (CCM), the nutrient solution essential for cell proliferation. Current formulations still rely heavily on animal-derived ingredients such as fetal bovine serum (FBS) [5,6], which not only entail high costs but also pose ethical and sustainability concerns, especially considering the large amounts required for large-scale CM production [7]. This explains why the development of sustainable and functional alternatives to conventional media is a research priority nowadays [8,9].

Furthermore, the use of animal-derived ingredients not only entails high costs but also represents an ethical limitation, a key principle of cellular agriculture [10]. The role of the CCM extends far beyond proliferation; it accompanies the cells throughout the entire production process, including advanced stages such as 3D-bioprinting, where cells are exposed to mechanical and oxidative stress [11]. Under these conditions, the ability of the CCM to support not only cell growth but also cell resilience becomes crucial. In this context, the design of CCM that enhance antioxidant activity plays a dual role: on the one hand, it strengthens cell resilience, and on the other, it allows the generation of functionally enriched products tailored to the needs of specific consumer groups, as well as the modulation of the nutritional quality of the final biomass [12]. This approach would promote the production of CM with an improved protein profile and enhanced functional properties, thus going beyond the simple replacement of FBS towards active customisation of cultured products.

Among the alternatives to animal-derived ingredients, particular attention should be given to solutions that ensure cell viability, proliferation, and differentiation while supporting environmental sustainability, such as plant-based products and food industry co-products [8,9,13,14]. Recent research has increasingly focused on co-product-derived protein hydrolysates and extracts as sustainable and functional supplements in serum-free or reduced-serum culture media for CM. Protein hydrolysates obtained from various plant sources, including corn and potato, have been shown to enhance muscle cell proliferation and differentiation under serum-free conditions, highlighting their potential as alternatives to animal-derived components in cell culture media [15]. In addition, plant-based agro-industrial residues, such as soybean and peanut meals, have been identified as low-cost, nutrient-rich resources capable of supplying essential amino acids and bioactive peptides while promoting circular economy principles [16,17]. Plant-derived hydrolysates have also been successfully incorporated into bioprinted CM constructs, demonstrating their multifunctional role in supporting cell growth and differentiation within advanced fabrication systems [18]. Collectively, these studies underscore the broad potential of plant-based co-products to serve as sustainable, functional, and ethically aligned ingredients for next-generation CCM formulations. Nevertheless, despite the growth of literature on plant-derived co-products, the exploration and valorization of animal-derived co-products for CM applications remain at an early stage.

Within this framework, marine co-products, including those derived from shrimp (*Penaeus* spp.) (SH) and blue crab (*Callinectes sapidus*) (BC) are of particular interest. These materials, often regarded as waste by the seafood processing industry [19], are rich in proteins, peptides, amino acids, and bioactive compounds such as chitin, collagen, and minerals [20,21,22]. When subjected to enzymatic hydrolysis, these co-products can yield hydrolysates and peptide fractions with high biological value, excellent digestibility, and documented antioxidant, antimicrobial, and growth-promoting properties [21]. Their recovery and valorization not only reduce the environmental impact associated with marine waste disposal [19], but also align with the principles of circular bioeconomy, transforming low-value co-products into nutritional sources for cell culture systems. In the context of cellular agriculture, such marine-derived hydrolysates may serve as sustainable and functional protein-rich ingredients, providing essential nitrogen sources and growth factors necessary for cellular proliferation while simultaneously enhancing antioxidant resilience under in vitro conditions

Complementary to these resources, plant-based ingredients, notably hemp (*Cannabis sativa* L.) seeds (HSs) and their derivatives, represent another promising class of alternative feedstocks. Hemp-based products, including hempseed protein (HP) and hemp flowers (HFs) as co-products, are characterized by a balanced amino acid profile, high polyunsaturated fatty acid content, and the presence of bioactive phytochemicals such as phenolic compounds and cannabinoids with antioxidant and anti-inflammatory potential [23,24,25]. These compounds can modulate cell metabolism and improve oxidative stability, which are critical parameters in optimizing the performance of cell-based production systems.

The integration of marine and plant-based co-products as functional components in CCM formulations could offer a novel and holistic approach to advancing cellular agriculture. By exploiting their nutritional density, bioactive potential, and sustainable origin, these ingredients can support cell growth and differentiation while contributing to a more resilient, circular, and ethically aligned cellular food production system. Therefore, exploring the biochemical composition, functional properties, and biological performance of these resources represents a crucial step toward the development of next-generation media designed for cell-based food production.

Considering the above, this study provides the first evaluation of HF, HS, HP, SH, and BC hydrolysates as CCM supplements for C2C12 cells, a well-established murine skeletal muscle myoblast line commonly used in the field of CM to study muscle cell proliferation and differentiation [26].

Cell viability was monitored at 24, 48, and 72 h, while the protein content was quantified both in the medium during proliferation and in the cell biomass after 72 h, to understand the extent to which the cell model (C2C12) was able to utilize these sustainable protein sources. Finally, considering the functional properties of the matrices, the antioxidant activity was evaluated both in the exhausted medium during culture and in the cell lysates (72 h) providing new insights into their potential to enhance both cell performance and the functional profile of CM. Overall, this study not only tests the feasibility of plant- and marine-derived protein hydrolysates as supplements for CCM, but also explores their potential to improve the nutritional and functional profile of CM, suggesting the possibility that they could contribute to the development of a sustainable, resilient, and health-promoting cellular agriculture.

## 2. Materials and Methods

### 2.1. Material

Given the low environmental impact and high nutritional and functional profile [18,19,20,21], hemp (*Cannabis sativa* L.) was selected among plant-based candidates, focusing on HF, HS, and commercial HP. As described in our previous work [24], HFs (*Cannabis sativa* L., variety Carmagnola) were provided by a local company (Cuneo, Italy). Manual harvesting (removal of cut plants) was followed by slow drying in a closed, ventilated environment, without direct light, until a moisture content of 12–14% was reached. Prior to analysis, HF samples were stored at room temperature (RT) in the dark. HSs (*Cannabis sativa* L., variety Futura) were purchased from a Czech company (Chrastice, Czech Republic). After collection, the seeds were dried at 40 °C until reaching a final moisture content of 7% and subsequently stored at 10–12 °C in the dark. Prior to each analysis, HS samples were stored at 4 °C in the dark. The HP was obtained from a commercial supplier.

Animal-derived raw materials: marine co-products, *Penaeus* spp. (SH) (composed of cephalothorax and abdominal parts) and *Callinectes sapidus,* blue crab (BC) (consisting of shells and legs), were collected from local commercial processing plants. Samples were transported to the laboratory in cold containers and stored at −20 °C until processing. Prior to analysis, samples were rinsed with water, dried in a ventilated oven at 60 °C for 72 h, and ground to a particle size of approximately 2 mm [21]. The material was then divided into three independent replicates (250 g each), vacuum-packed into plastic bags, and stored at −80 °C until analyses.

### 2.2. Methods

#### 2.2.1. Production of Plant- and Animal-Derived Hydrolysates

Plant- and animal-derived raw materials were processed to obtain protein hydrolysates following the procedure described by Messina et al. [21] and Arena et al. [27]. Briefly, co-products were resuspended in distilled water (1:10, *w*/*v*) with continuous stirring and pH monitoring. Protein hydrolysates were obtained using the bacterial protease Alcalase (≥2.4 U/g; Merck KGaA, Darmstadt, Germany). The enzyme was applied at an enzyme-to-substrate (E/S) ratio of 3% (*w*/*w*), under conditions optimized for each substrate. The pH was maintained by controlled addition of 5 N NaOH (Merck KGaA, Darmstadt, Germany) throughout the reaction.

The specific reaction conditions for each matrix (Table 1) were adopted based on established procedures: Ren et al. [28] for the plant matrices (HF, HS and HP), Messina et al. [21] for the SH matrix, and Arena et al. [27] for the BC matrix.

Enzymatic reactions were stopped by heating at 90 °C for 5 min. Hydrolysates were centrifuged at 7142× *g* for 15 min at 4 °C (Eppendorf 5430 R, Hamburg, Germany), and the resulting supernatants were lyophilized and stored at 4 °C until further analyses.

The Degree of Hydrolysis (DH%) was determined every 15 min using the following Equation (1):

*DH* (%) = (*V* × *M* × 100)/(*α* × *m_p_* × *h_tot_*) (1)

where *V* is the volume (mL) of sodium hydroxide consumed during hydrolysis; *M* the molarity of sodium hydroxide; α is the dissociation factor for oc-NH_2_ groups; *m_p_* the mass (g) of protein in the raw material introduced in the system; and *h_tot_* is the total number of peptide bonds in the protein samples [21,27,29].

#### 2.2.2. Antioxidant Activity of Hydrolysates

The antioxidant activity of the different hydrolysates determined with the 2,2-diphenyl-1-picrylhydrazyl (DPPH) and reducing power assay

For the antioxidant activity evaluated with DPPH, the protocol followed was that of Messina et al. [21]. Serial dilutions of the hydrolysate stock solution (30 mg/mL in water) were prepared to final concentrations of 0.25, 0.5, 1.0, 2.0, and 4.0 mg/mL. For each assay, 40 µL of sample was mixed with 160 µL of 100 µM DPPH in 96% ethanol (Merck KGaA, Darmstadt, Germany) and incubated in the dark at RT for 30 min. Absorbance was measured at 517 nm using a Multiskan -Sky Microplate Reader (Thermo Fisher Scientific™, Waltham, MA, USA). Gallic acid (Merck KGaA, Darmstadt, Germany) was used as a positive control.

All measurements were performed in six replicates.

The percentage of radical scavenging activity was calculated according to the following Equation (2):

(2) $$I\%=1-\left(\frac{A_{sample}}{A_{blank}}\right)\times 100$$

where *A_sample_* is the absorbance of the sample and *A_blank_* is the absorbance of the blank.

The antioxidant capacity was expressed as the mean IC_50_ ± SD, where IC_50_ is defined as the concentration required to reduce the initial absorbance by 50%.

The reducing power of the hydrolysates was determined at five concentrations using the spectrophotometric iron reduction method, as described by Abdelaleem and Elbassiony [30]. Hydrolysate solutions were mixed with 0.2 M phosphate buffer (pH 6.6) and 1% (*w*/*v*) potassium ferricyanide [K_3_Fe(CN)_6_] in a 1:1:1 (*v*/*v*/*v*) ratio and incubated at 50 °C for 20 min. An equal volume of 1% (*w*/*v*) trichloroacetic acid was added, and the mixture was centrifuged at 3000 rpm for 10 min. The upper layer was combined with distilled water and 0.1% (*w*/*v*) FeCl_3_ in a 1:1:2 (*v*/*v*/*v*) ratio, and absorbance was measured at 700 nm using a Multiskan-Sky Microplate Reader (Thermo Fisher Scientific™, Waltham, MA, USA). All reagents were purchased from Merck KGaA, Darmstadt, Germany.

The increase in absorbance of the reaction mixture reflected an increase in reducing power. The EC_50_ value (mg/mL), defined as the concentration of hydrolysate producing an absorbance of 0.5, was determined by linear regression analysis.

#### 2.2.3. Sample Preparation for Cell Culture

For cell culture experiments, lyophilized hydrolysates were resuspended in 45 mL of FBS-free medium to obtain a pH compatible with cell viability. Solutions were sterilized by filtration through a 0.2 µm membrane. To ensure a fair comparison between treatments, all hydrolysate solutions were standardized based on the sample with the lowest yield by dry weight (i.e., the HS hydrolysate), resulting in an initial stock concentration of 11.7 mg/mL for all treatments. From this stock, serial 1:2 dilutions were prepared up to 0.02 mg/mL and tested on the cells as described below.

#### 2.2.4. Cell Viability and Proliferation

The murine C2C12 skeletal muscle cell line (IZSLER, Brescia, Italy) was cultured for 9–18 passages in 75 cm^2^ flasks (Corning, Sigma-Aldrich, Milan, Italy) under standard conditions using a complete growth medium. The complete growth medium consisted of high-glucose DMEM (Sigma-Aldrich, Milan, Italy) supplemented with 10% FBS (Immunological Sciences, Rome, Italy), 2 mM L-glutamine (Sigma-Aldrich, Milan, Italy), and 1% penicillin–streptomycin (Immunological Sciences, Rome, Italy). Once the cells reached 70–80% confluence, they were detached with 5 mL of 1× trypsin–EDTA (Immunological Sciences, Rome, Italy), and viable cells were quantified using the trypan blue exclusion assay (Sigma-Aldrich, Milan, Italy) with a hemacytometer.

Following trypsinization, cells were seeded into 96-well plates (Corning, Sigma-Aldrich, Milan, Italy) at a density of 1.25 × 10^5^ cells/mL in complete growth medium, as previously described. After 24 h of incubation at 37 °C in a humidified atmosphere with 5% CO_2_, allowing for cell attachment and initial proliferation, the standard medium was replaced with medium containing different concentrations (11.7; 5.85; 2.93; 1.47; 0.73; 0.37; 0.18; 0.09; 0.05 and 0.02 mg/mL) of HF, HS, HP, SH, or BC (without any percentage of FBS), as specified above. Cultures were then maintained for an additional 24, 48, or 72 h. Cell viability was determined at each time point using the MTT assay (3-(4,5-dimethylthiazol-2-yl)-2,5-diphenyltetrazolium bromide) (Sigma-Aldrich, Milan, Italy) [31]. After the treatment period, the culture medium was replaced with 100 µL of MTT solution (0.25 mg/mL in 1× PBS) (Sigma-Aldrich, Milan, Italy) and cells were incubated for 2 h. The supernatant was then removed, and the formed formazan crystals were solubilized in an equal volume of dimethyl sulfoxide. Plate readings were performed using a BioTek Synergy HTX multimode microplate reader to determine cell viability (BioTek Instruments, Inc., Winooski, VT, USA) The resulting values were then normalized to the negative control (cells cultured in DMEM without FBS) and statistically compared with the positive control (cells cultured in complete growth medium).

At the end of the proliferation period, the optimal concentration for each matrix was determined and the different concentrations were compared in terms of cell viability using the MTT assay and through morphological analysis, as described in detail below.

#### 2.2.5. Cell Morphology

The concentrations showing the highest efficacy in supporting cell viability at 24, 48, and 72 h were selected for morphological analysis. Cells were seeded in 6-well plates (Corning, Sigma-Aldrich, Milan, Italy) at a density of 1.25 × 10^5^ cells/mL (total volume: 3 mL). At each time point (0, 24, 48, and 72 h), the CCM was removed and immediately stored at −80 °C for subsequent determination of protein concentration and antioxidant activity, as described below. Cells were then rinsed with 1× phosphate-buffered saline (PBS), and their morphology was assessed using an inverted light microscope equipped with a 10× objective lens (Optika, Bergamo, Italy). For each treatment group, four fields were examined, and the most representative images were included in the results. After image acquisition at 72 h, the cells were collected and immediately frozen at −80 °C for later analysis of protein content and antioxidant activity, as detailed below.

#### 2.2.6. Determination of Protein Concentration in Exhausted Cell Culture Media and Cell Biomass

As previously described, the initial stock and the exhausted CCM collected up to 72 h were used for protein concentration analysis. Simultaneously, the cell biomass pellet harvested after 72 h was pooled for each treatment and subsequently subjected to cell lysis. For total protein extraction, cell pellets were sonicated on ice using DU-32 digital ultrasonic bath (Argolab, Modena, Italy) applying 2–4 cycles of 10 s each, interspersed with 20 s pauses, with the amplitude set between 20 and 30% to achieve efficient lysis while preserving protein integrity in the presence of protease inhibitors.

Subsequently, the protein concentration of both the conditioned medium and cell lysates was determined using the bicinchoninic acid (BCA) Protein Assay Kit (Pierce™, Thermo Scientific™, Waltham, MA, USA, Cat. No. 23225), following the manufacturer’s protocol with minimal adaptations for small volumes. The samples and bovine serum albumin (BSA) standards at known concentrations were incubated with the BCA working reagent in test tubes for 30 min at 37 °C, a condition that allows the reduction of Cu^2+^ to Cu^1+^ by proteins and the formation of the coloured complex with BCA. The intensity of the resulting purple colour, proportional to the protein concentration, was measured by BioTek Synergy HTX multimode microplate reader at 562 nm. The protein concentration of the samples was calculated by interpolating the absorbance values on the BSA standard curve, performing at least three technical replicates for each sample. All reagents were kept at RT and the samples on ice until analysis to preserve protein integrity.

#### 2.2.7. Determination of Antioxidant Activity in Exhausted Cell Culture Media and Cell Biomass

As described, both the initial stock and the exhausted CCM were analysed for antioxidant activity, as was the cell biomass collected at 72 h. The exhausted CCM were analysed using the Ferric Reducing Antioxidant Power (FRAP) and DPPH assays.

For FRAP assay, the reagents used included ferric chloride hexahydrate (FeCl_3_·6H_2_O), ferrous sulphate (FeSO_4_), sodium acetate trihydrate, glacial acetic acid, and 2,4,6-tripyridyl-s-triazine (TPTZ), all purchased from Sigma Chemical Co. (St. Louis, MO, USA). The FRAP assay was performed according to the protocol of Abdelaleem and Elbassiony [30], with the following preparations:

(a) Acetate buffer (300 mM, pH 3.6): 2.69 g of sodium acetate trihydrate dissolved in 16 mL of glacial acetic acid, final volume brought to 1 L with distilled water.
(b) 2,4,6-tripyridyl-s-triazine (TPTZ) solution (10 mM): 31.2 mg of TPTZ dissolved in 10 mL of 40 mM HCl.
(c) FeCl_3_·6H_2_O solution (20 mM): 0.054 g dissolved in 10 mL of distilled water.

Ferrous sulphate was used as a reference for the calibration curve, prepared with six concentrations ranging from 0 to 1500 µM. The FRAP working reagent was prepared fresh by mixing 2.5 mL of TPTZ, 2.5 mL of FeCl_3_·6H_2_O and 25 mL of acetate buffer. For the analysis, 10 µL of sample was added to 300 µL of FRAP reagent, incubated for 20 min at RT in the dark, and the absorbance was read at 595 nm. Solvent controls (blank) were included. The results were expressed as mg FeSO_4_ equivalents per 100 g, considering at least three technical replicates for each biological replicate.

For the antioxidant activity evaluated with DPPH, the protocol followed was that of Prieto et al. [32] with some modifications. The DPPH stock solution (0.2 mM) was prepared by dissolving 39.4 mg of DPPH (Merck KGaA, Darmstadt, Germany) in methanol, bringing the volume to 500 mL in a calibrated flask. The solution was stored at 4 °C in the dark. The assay was conducted in 96-well plates, adding 100 µL of DPPH solution and 100 µL of appropriately diluted sample to each well. Controls included wells containing only DPPH (positive control) and only solvent (blank). The plates were incubated for 30 min at RT away from light, and then the absorbance was read at 517 nm with a microplate reader.

In parallel, the total antioxidant capacity of the cell pellets collected at 72 h was assessed using the FRAP assay, as previously described. Finally, the results were standardised according to the total protein content of the respective lysates, measured as described above. At least three technical replicates were performed for each sample, and the data were averaged for each biological replicate.

### 2.3. Statistical Analysis

Cell viability at 24, 48, and 72 h, as well as protein content and antioxidant activity in cell lysates at 72 h, were analyzed using one-way ANOVA followed by Tukey’s post hoc test for multiple comparisons (GraphPad Prism 9.3.1; GraphPad Software Inc., San Diego, CA, USA). Results are expressed as mean ± standard error of the mean (SEM) from at least three independent experiments. Statistical significance was set at a 95% confidence level (*p* ≤ 0.05). Correlations between antioxidant activity in CCMs and cell biomass after 72 h were evaluated using Pearson’s correlation coefficient, with significance determined at the same confidence level.

A principal component analysis (PCA) was carried out on three variables: cell viability, CCM FRAP and intracellular FRAP at 72 h, across six treatments (HF, HP, HS, SH, BC and 10% FBS). The PCA was conducted using STATISTICA software (version 8.0, StatSoft Inc., Tulsa, OK, USA).

## 3. Results

### 3.1. Production of Plant- and Animal-Derived Hydrolysates and Antioxidant Activities

**Figure 1:**
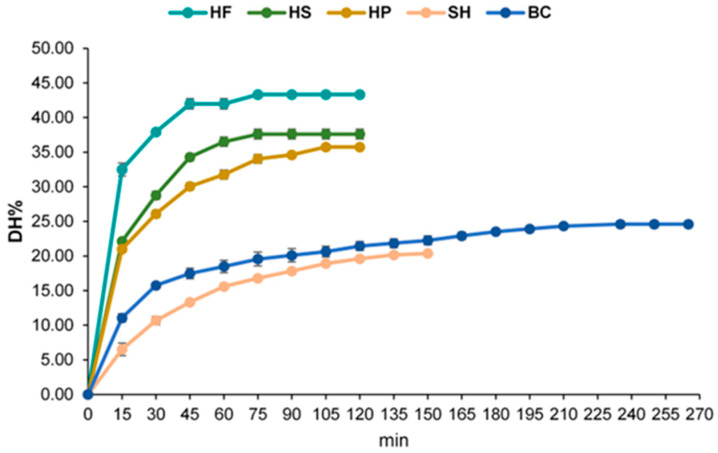
Degree of hydrolysis (% DH) for hemp flowers (HFs), hempseeds (HSs), hempseed protein (HP), and marine co-products (shrimp, SH; blue crab, BC) treated with Alcalase. Data are presented as mean ± SEM.

Protein hydrolysates were obtained by enzymatic hydrolysis using the bacterial protease Alcalase. Hydrolysis conditions were specifically adapted for each substrate matrix, as detailed in Table 1. Hemp-derived materials (HF, HS, and HP) and SH were processed at 60 °C and pH 8.5 for 120–150 min, BC was treated at 53 °C and pH 9.0 for 265 min. Under these optimized conditions, Alcalase promoted efficient hydrolysis across all matrices (Figure 1), with interesting antioxidant activity (Table 2). The Degree of Hydrolysis (DH%) values reported correspond to plateau conditions, defined as the stage at which further incubation did not result in a significant increase in hydrolysis.

The DH% values ranged from 20.38% (SH) to 43.34% (HF). The hemp-derived substrates generally exhibited higher susceptibility to Alcalase than the marine co-products. Specifically, HF reached the highest DH% at 43.34 ± 0.28%, followed by HS at 37.61 ± 0.71% and HP at 35.74 ± 0.15%. Regarding the marine co-products, BC hydrolysates achieved a DH% of 24.59 ± 0.50%, while the SH hydrolysates exhibited the lowest hydrolysis efficiency, with a DH% of 20.38 ± 0.12%. These results confirm that Alcalase is an effective protease for all tested substrates, demonstrating superior efficiency in terms of both hydrolysis rate and final DH% for the hemp-derived materials.

The antioxidant activities of the hydrolysates, as assessed by DPPH radical scavenging and reducing power assays, are shown in Table 2.

As reported, HF exhibited the highest DPPH radical scavenging activity (IC_50_ = 7.92 ± 1.01 mg/mL) and the strongest reducing power (EC_50_ = 30.56 ± 1.77 mg/mL), followed by HS (IC_50_ = 9.71 ± 1.52 mg/mL; EC_50_ = 37.44 ± 2.17 mg/mL), HP (IC_50_ = 10.67 ± 1.34 mg/mL; EC_50_ = 41.14 ± 2.38 mg/mL), and BC (IC_50_ = 11.39 ± 1.63 mg/mL; EC_50_ = 61.92 ± 7.33 mg/mL). SH showed the lowest antioxidant activity in both assays (IC_50_ = 50.98 ± 3.85 mg/mL; EC_50_ = 116.04 ± 12.44 mg/mL).

### 3.2. Effects of Hydrolysates on Cell Vitality and Morphology

**Figure 2:**
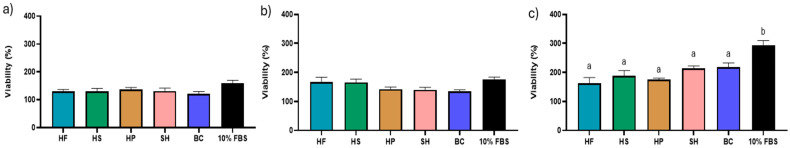
Comparison of the effects of HF (hemp flower), HS (hempseed), HP (hempseed protein), SH (shrimp) and BC (blue crab) hydrolysates on the viability of C2C12 muscle cells, assessed by MTT assay at 24 (**a**), 48 h (**b**) and 72 h (**c**). Values, expressed as %, are reported as average ± SEM, and normalized to the 0% FBS (Fetal bovine serum) control. Different superscript letters indicate statistically significant differences between treatments (*p* ≤ 0.05).

To allow for a fair comparison between treatments, all solutions were standardised based on the total amount of material obtained from the sample with the lowest yield after the hydrolysis process (HS), establishing an initial concentration of 11.7 mg/mL for all treatments. As reported in the supplementary material (Supplementary Figures S1–S5), the highest concentration tested (11.7 mg/mL) resulted in the most pronounced effects for HF, HS, HP, SH, and BC, with responses comparable to those observed with 10% FBS at 24, 48, and 72 h. Based on these results, 11.7 mg/mL was selected for each hydrolysate to systematically evaluate and compare cellular responses to different treatments at 24 h (Figure 2a), 48 h (Figure 2b) and 72 h (Figure 2c). After 24 h, no significant differences were observed among HF (130.97 ± 6.29%), HS (130.78 ± 10.09%), HP (138.88 ± 7.61%), SH (131.06 ± 10.91%), BC (121.43 ± 8.25%), and 10% FBS (158.55 ± 11.41%). A similar trend was observed at 48 h, where the hydrolysates (HF: 167.66 ± 15.44%; HS: 165.85 ± 10.90%; HP: 142.14 ± 7.52%; SH: 140.33 ± 8.07%; BC: 134.30 ± 5.46%) showed no significant differences compared to 10% FBS (175.77 ± 8.51%).

At 72 h, as shown in Figure 2c, the 10% FBS treatment (292.55 ± 20.47%) resulted in significantly higher cell viability (*p* ≤ 0.05) than all hydrolysates tested.

**Figure 3:**
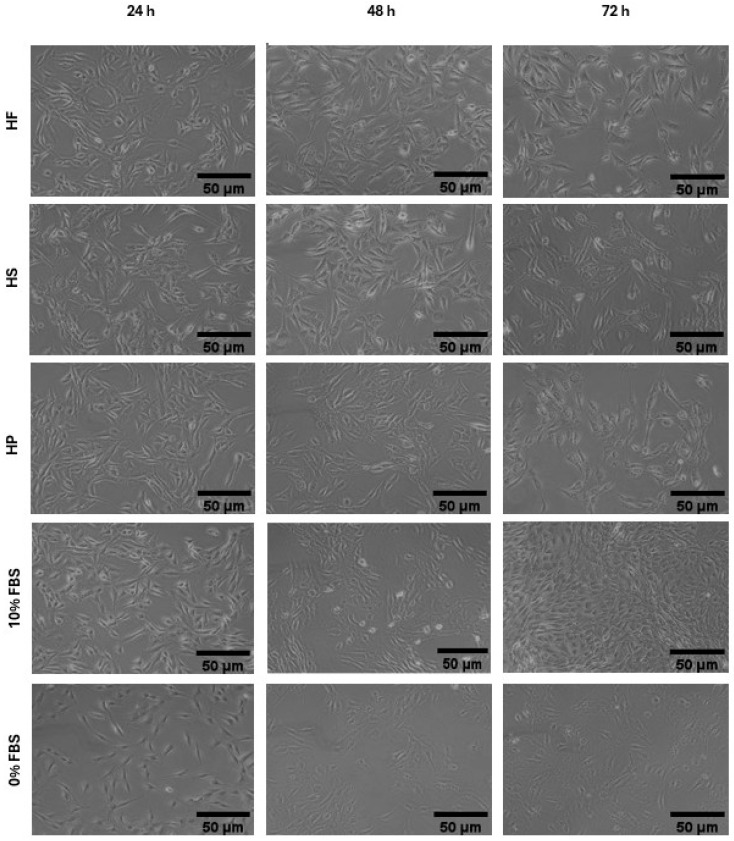
Morphology of C2C12 cells cultured in media supplemented with 11.7 mg/mL of HF (hemp flower), HS (hempseed), HP (hempseed protein) hydrolysates, 10% FBS (fetal bovine serum) and 0% FBS after 24 and 48 and 72 h. Images were acquired at 10× magnification.

**Figure 4:**
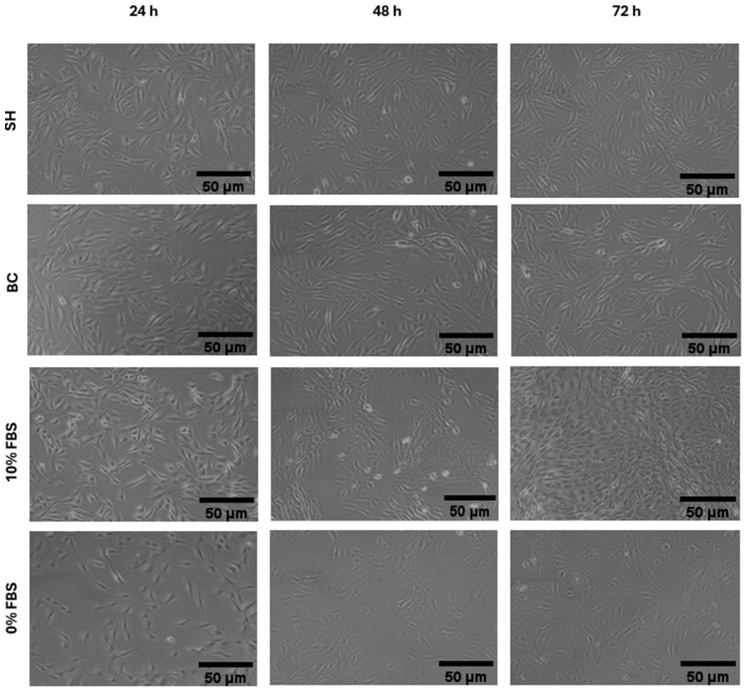
Morphology of C2C12 cells cultured in media supplemented with 11.7 mg/mL of SH (shrimp), BC (blue crab), 10% FBS (fetal bovine serum) and 0% FBS after 24 and 48 and 72 h. Images were acquired at 10× magnification.

Cell morphology of hemp-based products (Figure 3) and of marine co-products (Figure 4) was assessed by examining cell shape, adhesion, and distribution on the substrate at each proliferation time point. At 24 and 48 h (Figure 3 and Figure 4), all treatments preserved the typical elongated, fusiform morphology of C2C12 myoblasts, with effective substrate adhesion and a homogeneous distribution comparable to the positive control (10% FBS). In addition, cell density increased steadily from 24 to 48 h, and all treatments produced a higher density than the negative control (0% FBS) (Figure 3 and Figure 4). At 72 h, morphological observations partially confirmed the trends reported for cell viability in Figure 2c. Cells treated with 10% FBS exhibited high density and retained a characteristic myoblastic phenotype, marked by a fusiform shape, compact cytoplasm, and aligned orientation.

### 3.3. Evaluation of Protein Content in Cell Culture Media and in Cell Biomass

**Figure 5:**
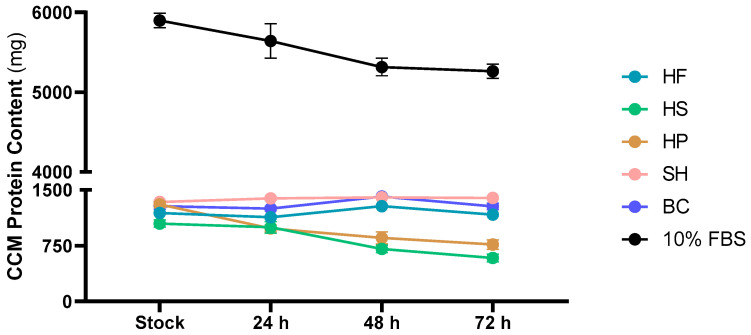
Protein content in the culture medium (CCM) in stock and after 24, 48 and 72 h of supplementation with HF (hemp flower), HS (hempseed), HP (hempseed protein), SH (shrimp) and BC (blue crab) hydrolysates, and with 10% FBS (Fetal bovine serum). The results were normalised with respect to the protein content of the control medium (0% FBS) and expressed in mg.

Figure 5 shows the protein content in the stock and in the exhausted CCM up to 72 h. As shown, the 10% FBS stock (5898.25 ± 91.08 mg) had higher protein content than all the hydrolysates. Similar values were reported for HF (1191.27 ± 40.31 mg), HS (1046.36 ± 48.98), HP (1305.52 ± 42.61 mg), SH (1337.89 ± 22.44 mg) and BC (1281.17 ± 42.84 mg). At the various time points, the protein content for each matrix decreased or remained constant up to 72 h, at which point 10% FBS (5262.73 ± 88.48 mg) confirmed higher values than the hydrolysates (1168.35 ± 9.39 mg; 585.21 ± 51.13; 765.78 ± 63.76; 1392.10 ± 13.38; 1270.19 ± 42.63 mg, respectively, for HF, HS, HP, SH and BC).

**Figure 6:**
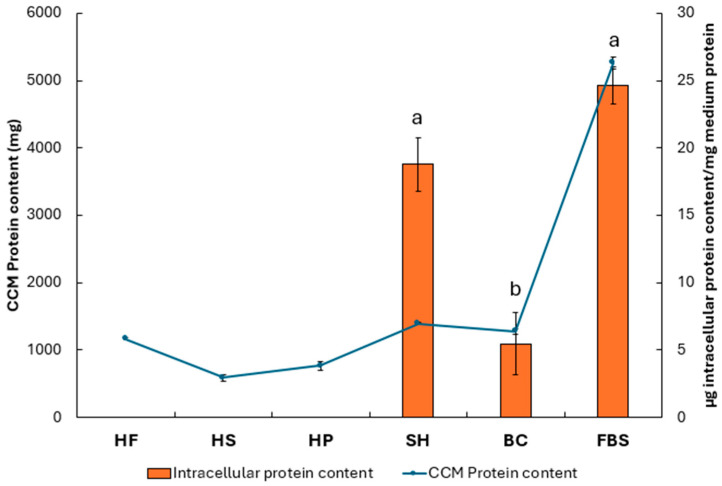
Quantification of intracellular protein content (orange bars, left *y*-axis) and protein content released into the cell culture medium (CCM; blue line, right *y*-axis) under different experimental conditions [HF (hemp flower), HS (hempseed), HP (hempseed protein), SH (shrimp) and BC (blue crab) hydrolysates, and with 10% FBS (Fetal bovine serum)]. Data are expressed as mean ± SEM. Superscript letters indicate statistically significant differences in intracellular protein content at 72 h (*p* < 0.05).

To evaluate the ability of cells to metabolize the proteins supplemented in the CCM, the intracellular protein content was quantified after 72 h and normalized with respect to the amount of protein present in the CCM stock (Figure 6). This normalization made it possible to estimate the amount of intracellular protein synthesized per mg of protein supplied. Cells treated with 10% FBS (24.66 ± 1.37 µg intracellular protein/mg medium protein) showed protein levels comparable to those observed with SH (18.79 ± 1.99 intracellular protein/mg medium protein), both significantly (*p* ≤ 0.05) higher than the BC (5.75 ± 2.32 intracellular protein/mg medium protein) treatment. Hemp-based hydrolysates were not included in the graph, as normalisation relative to the negative control yielded negative values. In parallel, Figure 6 also highlights the relationship between nutrient consumption from the CCM and cellular anabolism at 72 h.

### 3.4. Evaluation of Antioxidant Activity in Cell Culture Media and in Cell Biomass

**Figure 7:**
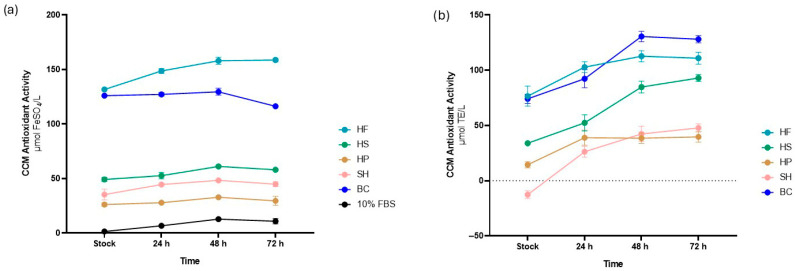
Antioxidant activity of cell culture media (CCM) supplemented with HF (hemp flowers), HS (hempseed), HP (hempseed protein), SH (shrimp), BC (blue crab), and 10% FBS (fetal bovine serum), evaluated using the FRAP (**a**) and DPPH (**b**) assays. Results (mean ± SEM) were normalized to the negative control (0% FBS) and expressed as µmol FeSO_4_/L for FRAP and µmol Trolox Equivalent/L (TE/L) for DPPH.

The antioxidant activity of CCMs enriched with hydrolysates was evaluated using FRAP (Figure 7a) and DPPH (Figure 7b) assays. The DPPH assay showed a gradual increase in antioxidant activity from the initial level in the stock up to 72 h for all samples analysed. A similar trend was also confirmed by the FRAP assay, although CCM supplemented with HP and BC maintained a relatively constant content at different time points.

In detail, the FRAP assay showed that the addition of HF (131.67 ± 2.32 µmol FeSO_4_/L) and BC (126.0 ± 0.5 µmol FeSO_4_/L) resulted in higher increase in antioxidant activity compared to HS (49.17 ± 1.87 µmol FeSO_4_/L), which in turn was higher than HP (26.17 ± 1.47 µmol FeSO_4_/L) and SH (35.33 ± 4.90 µmol FeSO_4_/L). All samples were higher than the positive control with 10% FBS (1.50 ± 0.22 µmol FeSO_4_/L). At 72 h, the same trend was observed.

The DPPH assay partially confirmed these findings, in agreement with the values reported in Table 2 and expressed as µmol Trolox equivalents per liter (µmol TE/L).

**Figure 8:**
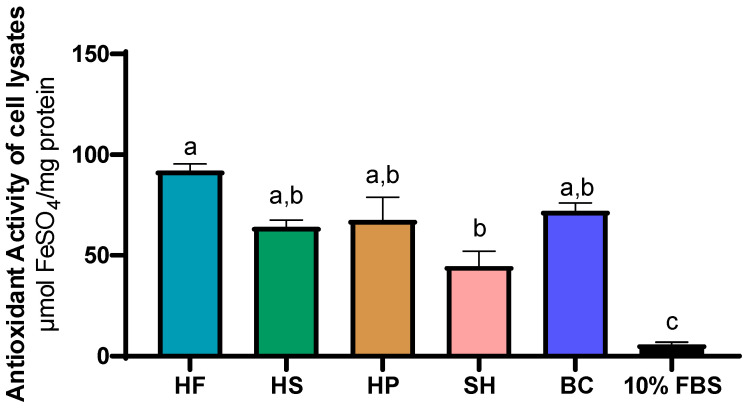
Antioxidant activity of cell lysates treated with HF (hemp flower), HS (hempseed), HP (hempseed protein), SH (shrimp), BC (blue crab) hydrolysates, and 10% FBS (fetal bovine serum), measured at 72 h. Results (mean ± SEM) were normalized to the negative control (0% FBS) and expressed as µmol FeSO_4_/mg protein. Different superscript letters indicate statistically significant differences between treatments (*p* ≤ 0.05).

Following the evaluation of antioxidant activity in CCM, the analysis was extended to cell lysate at 72 h. As shown in Figure 8, cells treated with 10% FBS (5.83 ± 1.12 µmol FeSO_4_/mg protein) showed significantly (*p* ≤ 0.05) lower antioxidant activity values than all other treatments. Treatment with HF (92.03 ± 3.23 µmol FeSO_4_/mg protein) showed numerically higher values, although comparable to those obtained with HS (64.07 ± 3.32 µmol FeSO_4_/mg protein), HP (67.56 ± 11.35 µmol FeSO_4_/mg protein) and BC (72.03 ± 3.91 µmol FeSO_4_/mg protein), but significantly (*p* ≤ 0.05) higher than SH (44.69 ± 7.42 µmol FeSO_4_/mg protein). No significant differences were found between HS, HP, BC and SH.

**Figure 9:**
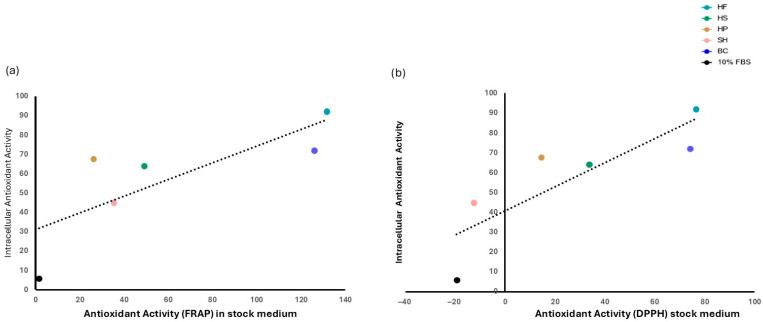
Correlation between intracellular antioxidant activity measured at 72 h and antioxidant activity present in the culture medium at stock, assessed using the FRAP (**a**) and DPPH (**b**) assays. Correlation significance was calculated using a 95% confidence interval.

To verify whether the antioxidant activity present in the stock CCM was correlated with intracellular activity at 72 h, a correlation analysis was performed. As shown in Figure 9b, intracellular antioxidant activity showed a strong and significant (*p* ≤ 0.05) correlation with that of the initial CCM measured using the DPPH assay (Pearson’s r = 0.94). A similar trend was also observed with the FRAP assay (Figure 9a) (Pearson’s r = 0.79), although in this case the correlation was not statistically significant.

### 3.5. Multivariate Analysis: Principal Component Analysis (PCA)

**Figure 10:**
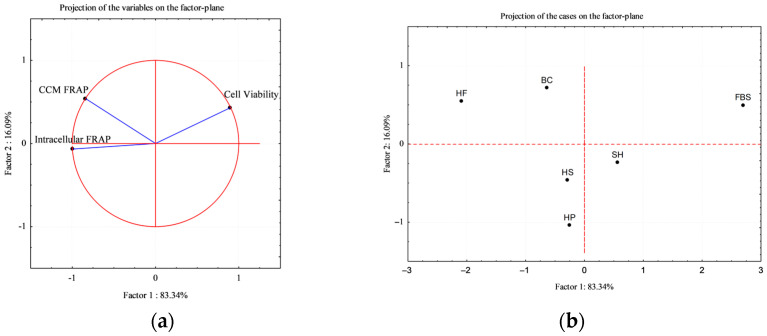
PCA obtained from the correlation of three variables (cell viability, CCM FRAP, and intracellular FRAP at 72 h) across six cases [HFs (hemp flowers), HS (hempseed), HP (hempseed protein), SH (shrimp) and BC (blue crab)]. (**a**) Correlation circle of variables; (**b**) Factor plane showing the separation of treatments.

To better visualize the correlation among cases vs. some variables, Principal Component Analysis (PCA) was applied. CMM FRAP and intracellular FRAP where selected for the correlation circle, as key variables for the study (Figure 10a); the factor plane (Figure 10b) shows the positioning of the treatments (cases) in relation to the variables.

The analysis revealed that the first two principal components explained more than 90% of the total variance (Factor 1: 83.34%; Factor 2: 16.09%), indicating that most of the variability in the dataset can be effectively represented in a two-dimensional space.

As it is shown in projection of the cases (b), the FBS control is distinctly separated from the other experimental cases and results positively correlated with cell viability variable; on the other side, the hydrolysate-supplemented media (HF, HS, H, SH, and BC), result positively correlated with both intrinsic antioxidant power of the hydrolysates (CMM FRAP) and intracellular antioxidant power (FRAP), attesting the prominent role of the antioxidant power on the composition of the experimental case study. Overall, PCA confirmed the trends observed in univariate analyses, and provided a concise multivariate visualization of the distinct biological behavior induced by sustainable protein hydrolysates compared to conventional serum supplementation.

## 4. Discussion

One of the main obstacles to the industrial development of CM is the high demand for CCM. As reported by Hubalek et al. [7], the production of 1 kg of CM requires approximately 20 L of media, with consequent economic, environmental and ethical implications. The current reliance on animal-derived ingredients, particularly FBS, ensures optimal cell growth, but is unsustainable on a large scale due to high costs, ethical limitations and standardization issues [33]. In this context, the search for sustainable alternatives that can replace or reduce the use of animal ingredients during the proliferation phase is a strategic priority [8,34,35]. At the same time, the formulation of CCM should also aim to support the production of animal-derived products with high nutritional and functional value, which is crucial both for consumer acceptance and for delivering high-quality products to the market and therefore needs to be further optimized for this purpose.

In this study, hydrolysates from both plant and marine-derived matrices were evaluated as potential CCM supplements to support short-term C2C12 cell growth. The objective was not necessary to exceed the performance of 10% FBS, the gold standard, but to assess the ability of these sustainable matrices to maintain cell viability.

Hydrolysis conditions were specifically adapted for each substrate (Table 1) and Alcalase efficiently hydrolyzed all matrices, with DH% values ranging from 20.38% (SH) to 43.34% (HF) (Figure 1). BC hydrolysates reached a DH% of 24.59 ± 0.50%, confirming that crustacean co-products can be successfully valorized into bioactive protein hydrolysates, aligning with our previous studies [34,35].

For the hemp-derived fractions, the DH% values achieved (35.74–43.3%) are within the range reported to yield highly bioactive peptides. For example, Ren et al. [28] demonstrated that Alcalase treatment of HP at a DH of 27.24% efficiently produced α-glucosidase inhibitory oligopeptides. Similarly, hemp-derived protein hydrolysates have been reported to exert biological effects in mammalian cell models, including the modulation of cell proliferation and oxidative stress responses, as reported by Juárez-Cruz et al. [36]. The high DH% observed in plant-based matrices, particularly HF (43.3%), suggests an extensive release of low and medium molecular weight peptides, which are typically associated with enhanced bioactivity [37].

The antioxidant capacity of these hydrolysates is closely related to their amino acid composition, peptide sequence, and molecular weight distribution [21,27,37]. In the present study, HF hydrolysates demonstrated the highest antioxidant activity, as indicated by the lowest IC_50_ for DPPH radical scavenging (7.92 ± 1.01 mg/mL) and the lowest EC_50_ for Reducing Power (30.56 ± 1.77 mg/mL). These results suggest that the strong antioxidant activity of HF may be attributed to the release of short-chain peptides, which are more sterically accessible and therefore more effective in scavenging free radicals. Regarding the marine-derived matrices, BC and SH also showed a significant antioxidant activity, consistent with previous studies on crustacean co-products. In these matrices, amino acids such as histidine, tyrosine, methionine, and cysteine are known to enhance antioxidant activity through mechanism including hydrogen donation, electron transfer, or metal ion chelation [21,27,37]. Overall, these findings suggest that BC and SH derived protein hydrolysates represent promising source of bioactive compounds and could be used as functional ingredients.

Although a detailed characterization of molecular weight distribution was beyond the scope of this research, the combined evidence of efficient hydrolysis, antioxidant capacity, and maintenance of cell viability suggests that both hemp- and marine-derived hydrolysates are promising, sustainable CCM supplements, capable of providing nutritional and functional support.

The results obtained show that the objective was achieved at least up to 48 h (Figure 2a,b) of culture, a significant milestone considering that the doubling time of muscle cells in vitro, under conditions intended for CM production, has been estimated to be between 24 and 40 h [38]. The maintenance of a positive effect up to this time threshold appears to be associated with the availability of essential amino acids released by hemp-based products, as well as by SH and BC protein hydrolysates. It is widely recognized that essential amino acids play a crucial role in stimulating protein synthesis [39], although the molecular mechanisms responsible for these effects remain unclear. According to Xu et al. [39], essential amino acids, especially branched-chain ones, not only act as indispensable precursors for protein anabolism but also exert a direct intracellular signalling action, promoting the activation of biosynthetic pathways involved in muscle metabolism. On the one hand, they act as the main transporters of amino nitrogen and, in addition, they are powerful direct stimulators of muscle protein synthesis through the activation of key pathways such as mTORC1 [40]. As reported in the literature, hemp-based products, SH and BC, are characterized by an amino acid profile rich in this fraction [41,42,43,44]. For this reason, the presence of these amino acids, in addition to providing metabolic substrates, may therefore have contributed to supporting the cell growth and proliferation processes observed up to 48 h.

Despite this, analysis after 72 h of culture showed that the best results were obtained in conditions containing 10% FBS (Figure 2c). This data appears consistent with the complex nature of the metabolic requirements of muscle cells. In addition to sources of nitrogen and amino acids, cells require an adequate energy supply, as well as growth factors, hormones, and signaling molecules that orchestrate cell proliferation and survival processes. These components are abundant in FBS [45], while, as expected, they are found in much lower concentrations in protein hydrolysates. However, all treatments with hydrolysates (HF, HS, HP, SH and BC) supported cell growth, with no statistically significant differences between them, but still in a more efficient way in comparison to the negative control (100%).

The most significant differences between the hydrolysates emerge from morphological analysis (Figure 3 and Figure 4). Cells treated with hemp-based hydrolysates (Figure 3) showed an altered phenotype, characterized by larger cells and voluminous nuclei, suggesting a slowdown in the cell cycle or a response to metabolic and genotoxic stress [46]. This is most probably related to the presence of phenolic compounds (phenolic acids, flavonoids and tannins) which, when released and solubilized during hydrolysis, could compromise the vitality of muscle cells in the long term, as already demonstrated by Chen et al. [47] in smooth muscle cells. Added to this is the presence of anti-nutritional factors typical of the plant matrix, such as phytates, known for their ability to chelate essential cations (Zn^2+^, Fe^2+^, Mg^2+^, Ca^2+^) and reduce the availability of enzymatic cofactors essential for DNA replication and protein synthesis [23]. Tannins can also interact with proteins and compromise their bioavailability, while, although in smaller quantities than in other plants, saponins and protease inhibitors could contribute to modifying membrane permeability or interfering with intracellular digestive and metabolic processes [23]. On the contrary, cells treated with marine hydrolysates (SH and BC) (Figure 4) had a morphology more similar to that observed in the presence of FBS, characterized by regular cells and nuclei of physiological size. This result can be attributed not only to the absence of anti-nutritional factors typical of plant-based matrices, but also to the more favourable amino acid profile of marine hydrolysates, which contain high levels of glycine, proline, hydroxyproline, arginine and, in particular, taurine, which is completely absent in plant sources [44]. These amino acids not only act as anabolic substrates for protein synthesis, but also play a crucial regulatory role, contributing to the stability of the extracellular matrix, the maintenance of osmotic homeostasis and the activation of signaling pathways involved in cell proliferation [44]. The availability of these components allows cells to maintain a cytoskeletal and nuclear organization closer to the physiological one, supporting proliferation without inducing the morphological alterations seen in treatments with hemp-based hydrolysates.

To evaluate protein absorption at the cellular level (Figure 6), the trend in protein content in the CCM was analyzed from the stock to the exhausted medium after 72 h (Figure 5). The analysis revealed differences between the protein matrices tested.

In CCM containing animal proteins, such as SH and BC, the overall protein concentration in the medium remained largely stable over time (Figure 5). This stability reflects the chemical resistance of these proteins to spontaneous degradation or interactions with other components of the medium, due to their complex three-dimensional structure and the presence of disulphide bridges [48]. However, a fraction of these proteins is still internalized by the cells. The apparent constancy of the concentration in the medium reflects a balance between cellular uptake and the intrinsic resistance of the remaining proteins to degradation, with a minimal net reduction over time, a behaviour confirmed by intracellular analysis at 72 h (Figure 6).

Similarly, CCM supplemented with HF also showed a stable protein concentration; however, the negative intracellular protein content (Figure 6) indicates limited biological availability. This phenomenon is probably related to the complexity of the plant matrix and the presence of fibers, polyphenols or other secondary metabolites that can form complexes with proteins, reducing their solubility and assimilation [49].

In CCM supplemented with 10% FBS, HS and HP, a decrease in protein concentration was observed over time. In the case of 10% FBS, this reduction reflects active consumption by cells: FBS proteins, which are highly soluble and easily hydrolysable [45], are broken down into free amino acids and peptides by extracellular proteases and rapidly internalized via specific transporters. This process explains the simultaneous increase in intracellular content, confirming the efficiency of these proteins as a source of nutrients for protein synthesis and cellular metabolism, hence 10% FBS remains the gold standard in supporting cell viability and proliferation [50]. For plant matrices such as HS and HP, however, the decrease in protein in the medium does not translate into significant intracellular accumulation. This, most probably, suggests that the observed reduction is due in part to chemical degradation or precipitation, rather than actual assimilation. Limited internalization can be attributed to the presence of anti-nutritional factors, such as protease inhibitors, lectins and polyphenols, which reduce the biological availability of protein and interfere with cellular metabolism [49].

At the mechanical level, protein assimilation depends on digestibility, solubility and accessibility of the protein matrix [51,52]. Soluble proteins that are not bound to complex matrices are hydrolyzed into peptides and free amino acids, internalized via endocytosis or specific transporters, and incorporated into intracellular protein synthesis. In contrast, proteins bound to fibers, phenolic compounds, or complex plant structures show reduced intracellular absorption [49].

These results highlight how the mere presence of proteins in the CCM does not guarantee their metabolic availability; digestibility, biological stability and possible interference from secondary compounds must also be considered [51]. The decline observed in the medium, as in the case of 10% FBS, is a positive indicator of active consumption, while plant proteins, although present or stable in the medium, show limited intracellular assimilation. To increase their availability [51], strategies such as enzymatic pretreatments, matrix fractionation or targeted chemical modifications may be necessary [53].

However, treatments with SH and BC induced similar cell morphology, albeit with lower overall cell density. In contrast, hemp-based hydrolysates led to obvious morphological alterations, including reduced cell density, increased cell size and prominent nuclei, characteristics indicative of a less favourable physiological and proliferative state. These observations are consistent with the results reported by Batish et al. [14]. In this context, Batish et al. [14] evaluated protein hydrolysates derived from marine invertebrates, including bivalve molluscs (oysters and mussels) and marine worms, as supplements in low-serum culture media. These marine-derived hydrolysates were shown to support cell viability and proliferation, confirming their suitability as alternative sources of nitrogen and peptides for animal cell culture. Although these materials were not co-products of the food industry, their application as a serum reduction strategy rather than a complete serum replacement yielded encouraging results, highlighting their potential relevance for CM production. In the search for animal-based ingredients that are more sustainable and ethically acceptable than FBS, insect-based alternatives have also been explored [14,15]. Insect protein hydrolysates obtained from species such as *Tenebrio molitor* and *Hermetia illucens* have been shown to promote cell growth under reduced serum conditions, confirming their potential as alternative protein sources for cell farming [5,14,15]. However, unlike protein sources derived from established agri-food supply chains, insect-based ingredients generally require the implementation of dedicated production systems, including insect rearing, harvesting and downstream biomass processing. This additional upstream step could increase both the cost and complexity of the process, particularly in the context of large-scale applications [54,55].

Finally, all these data highlight the importance of integrating protein content measurements in the CCM with intracellular analysis, as monitoring the medium alone can be misleading. The combination of chemical stability, digestibility and biological availability is essential for designing optimized culture media, especially when using alternative and sustainable protein sources. Understanding the mechanisms that regulate protein availability and assimilation, as well as any saturation or toxicity effects in prolonged cultures, is crucial for developing effective cell nutrition strategies and biotechnological applications, including CM.

Another factor to consider for potential industrial applications is the stability and cost-effectiveness of the supply of plant- and marine-derived co-products. The availability of these co-products can vary seasonally and geographically, and large-scale enzymatic hydrolysis requires investment in equipment, enzyme and process control. Although techniques such as ultrafiltration to obtain low-molecular-weight peptides, potentially increase bioactivity, they are resource and time-intensive. In this context, the use of total hydrolysates is simpler, more economical approach. Future studies should therefore focus on ensuring a stable supply of raw material and optimizing production costs for large-scale applications, particularly in the context of cultured meat production.

At the same time, as shown in Figure 7, antioxidant activity in the CCM was observed, which is a key parameter for assessing the ability of hydrolysates to provide functional properties to cell biomass. The analysis showed a gradual increase in antioxidant activity from the stock up to 72 h, both in FRAP and DPPH assays. This increase, which was particularly evident for the matrices studied, can be attributed both to the constant release of peptides into the CCM and to the action of extracellular proteases, which break down large protein chains into smaller fragments, thereby increasing their functional activity [56].

In detail, CCM supplemented with HF, HS, HP, SH and BC showed different absolute values, all of which were higher than those observed in conventional media containing 10% FBS, confirming the findings of Leist et al. [57] and Lewinska et al. [58], who highlighted the limited antioxidant functionality of standard media, even when supplemented with FBS. The differences in the increase in antioxidant activity can be attributed to the nature of the matrix considered. HF showed higher absolute values than the other samples. This effect is probably due not only to the presence of bioactive peptides, but also to the higher concentration of secondary compounds, such as polyhydroxy flavonoids, lignanamides and hydroxylated phenolic acids, which are more abundant in this fraction of the plant than in HS and are known for their high antioxidant activity [24,25].

The significant antioxidant capacity of the hemp-derived fractions is consistent with previous research demonstrating that enzymatic hydrolysis of hemp protein isolate by Alcalase effectively yields hydrolysates with strong DPPH radical scavenging, Fe^2+^ chelating, and reducing power [59].

At the same time, the HS hydrolysate showed higher values than HP, confirming the active role of secondary compounds together with functional peptides [23]. However, these same compounds may have contributed to a slowdown in cell proliferation, as already observed.

As for marine matrices, BC showed high antioxidant activity in FRAP and DPPH assays, mainly due to low molecular weight peptides rich in histidine, cysteine and taurine [60,61]. These peptides act both as electron donors and radical scavengers, thanks to thiol groups and aromatic residues capable of stabilizing free radicals. Free amino acids typical of crustaceans, such as taurine, cysteine, methionine, glycine and alanine, also contribute synergistically to antioxidant activity [60,61]. In addition, lipophilic carotenoids such as astaxanthin and chitin derivatives such as chito-oligosaccharides can further enhance the scavenger effect and metal chelation capacity, contributing to the stability of the antioxidant activity observed at 72 h [60].

Although SH has a very similar peptide and amino acid profile and comparable antioxidant molecule content in its exoskeleton [62], the values observed are still lower than those of BC. This marked difference, which is difficult to explain, does not necessarily reflect the intrinsic quality of the animal, but is likely to be determined by upstream factors, such as diet and primary matrix treatment. Considering the crucial role of astaxanthin and other carotenoids as antioxidants, it is important to remember that, as reported by Abd-El Ghani et al. [63], aquatic animals are unable to synthesize them independently but accumulate them through their diet. Consequently, the differences in antioxidant values between SH and BC may depend more on the nutritional profile and processing of co-products than on the intrinsic characteristics of the animal. This represents, as highlighted by Vastolo et al. [64], a critical aspect common to all food industry co-products.

Overall, the increase in antioxidant activity observed in the medium after 72 h can be attributed to the stability and accumulation of bioactive peptides, as well as the contribution of antioxidant metabolites secreted by the cells. These results indicate that both plant and marine hydrolysates are promising sources of direct and indirect antioxidants, which is particularly relevant in the context of CM. The concomitant presence of antioxidant activity and protein residues in the medium opens the prospect of circular use of CCM, improving the stability and quality of the culture and potentially allowing the medium to be recycled [65] for the growth of protein biomass-producing microorganisms, integrating economic and environmental sustainability, as already demonstrated, albeit on a pilot scale, by Haraguchi et al. [66].

This finding is confirmed by intracellular antioxidant activity (Figure 8). At 72 h, cells treated with protein hydrolysates showed a significant increase in reducing power compared to the FBS control, indicating that these compounds are not only biovailable but also capable of stimulating various intracellular antioxidant pathways. This highlights a direct link between the antioxidant compounds present in the medium and those detected within the cells (Figure 9). The increase in antioxidant power observed in cellular biomass suggests that plant and marine hydrolysates not only act synergistically to protect cells from oxidative stress, but also offer concrete opportunities to customize products, enhancing the functional profile of the final product [12,67].

## 5. Conclusions

This study evaluated the potential role of plant- and marine-derived protein hydrolysates obtained from food industry co-products as supplements for cell culture media (CCM). The results showed that, at short incubation times, hydrolysate-based treatments supported C2C12 myoblast viability at levels comparable to 10% fetal bovine serum (FBS). At 72 h, although FBS induced the highest proliferation, shrimp (SH) and blue crab (BC) preserved a more physiological myoblastic morphology and exhibited intracellular protein accumulation comparable to FBS-treated cells. In contrast, hemp-derived hydrolysates resulted in morphological alterations and limited intracellular protein utilization, underscoring the importance of protein bioavailability rather than mere presence in the culture medium. Moreover, all hydrolysates significantly enhanced intracellular antioxidant activity compared with FBS, suggesting potential functional effects on the resulting cell biomass. Overall, these findings highlight the relevance of food industry co-product-derived protein hydrolysates as promising CCM ingredients for cell proliferation. Future studies should focus on optimizing hydrolysate composition, improving long-term culture performance, and assessing scalability and regulatory aspects to support their potential implementation in industrial CM production.

## Figures and Tables

**Table 1:** Reaction conditions of HFs (Hemp flowers), HS (hempseed), HP (hempseed protein), SH (shrimp) and BC (blue crab).

Substrate	T°C	pH	Time (min)
HF	60	8.5	120
HS	60	8.5	120
HP	60	8.5	120
SH	60	8.5	150
BC	53	9	265

**Table 2:** DPPH Radical scavenging activity (IC 50) and Reducing power (EC 50) (mg/mL of extract) of HF (hemp flower), HS (hempseed), HP (hempseed protein), SH (shrimp) and BC (blue crab) hydrolysates. Data are expressed as mean ± standard deviation (*n* = 6).

Samples	DPPH Radical Scavenging Activity (IC 50 mg/mL of Extract)	Reducing Power (EC 50 mg/mL of Extract)
HF	7.92 ± 1.01	30.56 ± 1.77
HS	9.71 ± 1.52	37.44 ± 2.17
HP	10.67 ± 1.34	41.14 ± 2.38
SH	50.98 ± 3.85	116.04 ± 12.44
BC	11.39 ± 1.63	61.92 ± 7.33

## Data Availability

The original contributions presented in this study are included in the article/Supplementary Materials. Further inquiries can be directed to the corresponding authors.

## Supplementary Materials

The following supporting information can be downloaded at: https://www.mdpi.com/article/10.3390/foods15020352/s1, Figure S1: Effects of HF (hemp flower) hydrolysate on the viability of C2C12 muscle cells, assessed by MTT assay at 24 h (a), 48 h (b), and 72 h (c). Figure S2: Effects of HS (hempseed) hydrolysate on the viability of C2C12 muscle cells, assessed by MTT assay at 24 h (a), 48 h (b), and 72 h (c). Values are expressed in %, normalized to the 0% FBS (Fetal bovine serum) control and compared with 10% FBS. (*) indicate statistically significant differences (*p* ≤ 0.05) relative to 10% FBS. Figure S3: Effects of HP (hempseed protein) hydrolysate on the viability of C2C12 muscle cells, assessed by MTT assay at 24 h (a), 48 h (b), and 72 h (c). Values are expressed in %, normalized to the 0% FBS (Fetal bovine serum) control and compared with 10% FBS. (*) indicate statistically significant differences (*p* ≤ 0.05) relative to 10% FBS. Figure S4: Effects of SH (shrimp) hydrolysate on the viability of C2C12 muscle cells, assessed by MTT assay at 24 h (a), 48 h (b), and 72 h (c). Values are expressed in %, normalized to the 0% FBS (Fetal bovine serum) control and compared with 10% FBS. (*) indicate statistically significant differences (*p* ≤ 0.05) relative to 10% FBS. Figure S5: Effects of BC (Blue Crab) hydrolysate on the viability of C2C12 muscle cells, assessed by MTT assay at 24 h (a), 48 h (b), and 72 h (c). Values are expressed in %, normalized to the 0% FBS (Fetal bovine serum) control and compared with 10% FBS. (*) indicate statistically significant differences (*p* ≤ 0.05) relative to 10% FBS.

## Abbreviations

The following abbreviations are used in this manuscript:

BC	Blue Crab
CM	Cultured Meat
CCM	Cell Culture Media
FBS	Fetal Bovine Serum
HF	Hemp Flower
HS	Hempseed
HP	Hempseed Protein
SH	Shrimp
