# Supplementary material for: Valorization of Hemp, Shrimp and Blue Crab Co-Products as Novel Culture Media Ingredients to Improve Protein Quality and Antioxidant Capacity of Cultured Meat in Cell-Based Food Applications
Source: Foods. 2026 Jan 18;15(2):352. doi: 10.3390/foods15020352 (PMC12840615; doi:10.3390/foods15020352)
Supplement: Supplementary file 1 [file foods-15-00352-s001.zip › foods-4032841-supplementary.pdf]

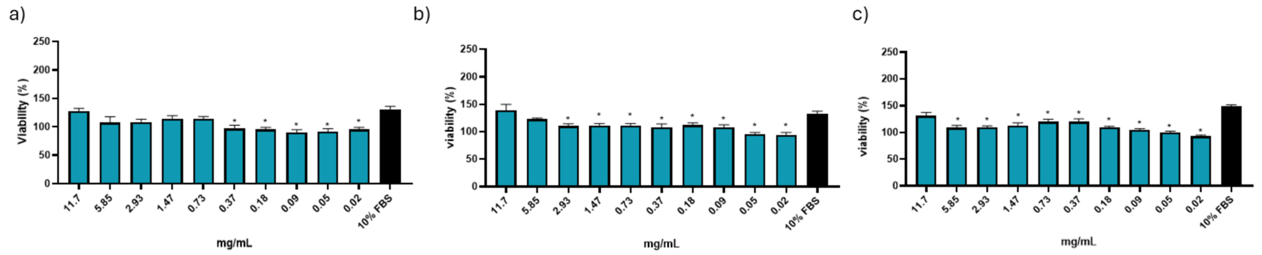

Supplementary Figure S1. Effects of HF (hemp flower) hydrolysate on the viability of C2C12 muscle cells, assessed by MTT assay at 24 h (a), 48 h (b), and 72 h (c). Values are expressed in %, normalized to the 0% FBS (Fetal bovine serum) control and compared with 10% FBS. (*) indicate statistically significant differences ($p \leq 0.05$) relative to 10% FBS.

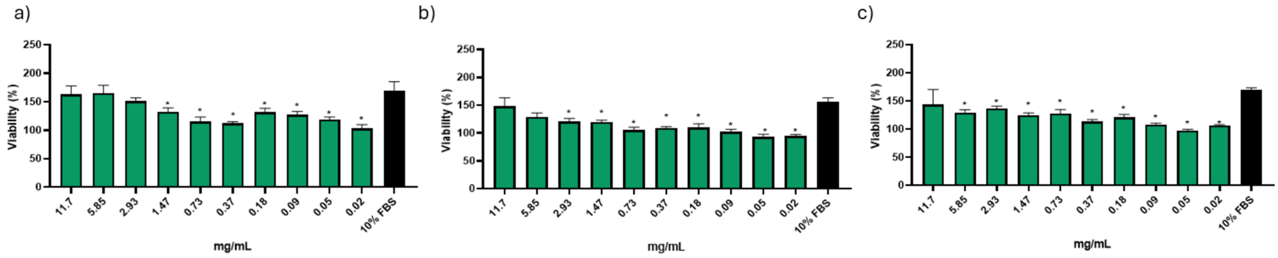

Supplementary Figure S2. Effects of HS (hempseed) hydrolysate on the viability of C2C12 muscle cells, assessed by MTT assay at 24 h (a), 48 h (b), and 72 h (c). Values are expressed in %, normalized to the 0% FBS (Fetal bovine serum) control and compared with 10% FBS. (*) indicate statistically significant differences ($p \leq 0.05$) relative to 10% FBS.

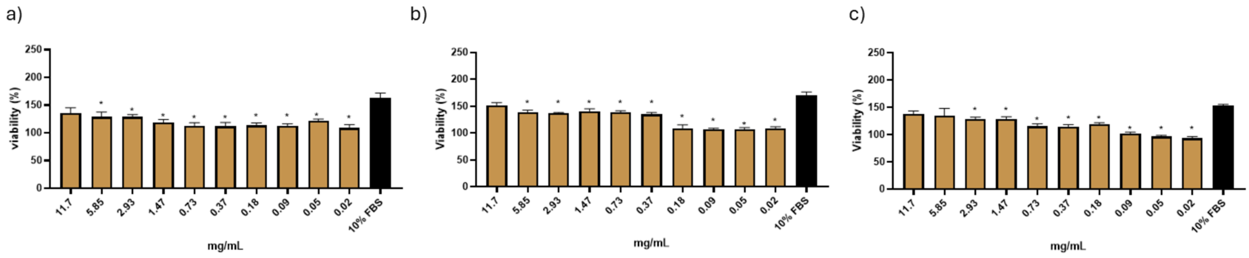

Supplementary Figure S3. Effects of HP (hempseed protein) hydrolysate on the viability of C2C12 muscle cells, assessed by MTT assay at 24 h (a), 48 h (b), and 72 h (c). Values are expressed in %, normalized to the 0% FBS (Fetal bovine serum) control and compared with 10% FBS. (*) indicate statistically significant differences (*p* ≤ *0.05*) relative to 10% FBS.

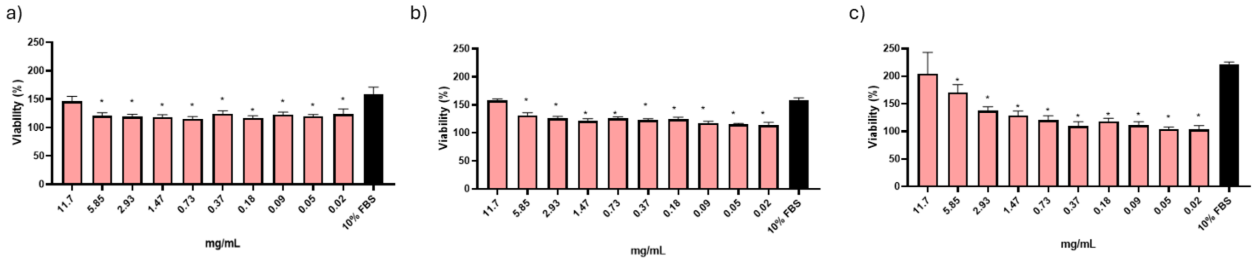

Supplementary Figure S4. Effects of SH (shrimp) hydrolysate on the viability of C2C12 muscle cells, assessed by MTT assay at 24 h (a), 48 h (b), and 72 h (c). Values are expressed in %, normalized to the 0% FBS (Fetal bovine serum) control and compared with 10% FBS. (*) indicate statistically significant differences (*p* ≤ *0.05*) relative to 10% FBS.

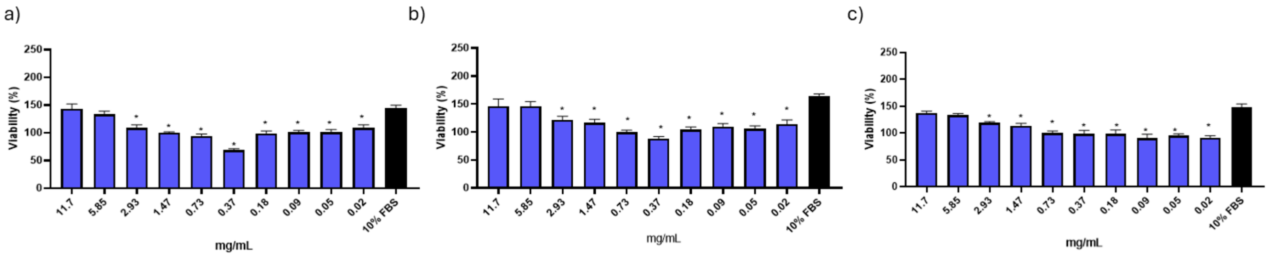

Supplementary Figure S5. Effects of BC (Blue Crab) hydrolysate on the viability of C2C12 muscle cells, assessed by MTT assay at 24 h (a), 48 h (b), and 72 h (c). Values are expressed in %, normalized to the 0% FBS (Fetal bovine serum) control and compared with 10% FBS. (*) indicate statistically significant differences ($p \leq 0.05$) relative to 10% FBS.
